# Electronic Clinical Decision Support System for Stroke Risk Screening in Patients With Atrial Fibrillation in Mental Health Care: Mixed Methods Study

**DOI:** 10.2196/66428

**Published:** 2025-08-06

**Authors:** Dina Farran, Hou Wang Cheang, Juliana Onwumere, Mark Ashworth, Fiona Gaughran

**Affiliations:** 1 Department of Psychosis Studies, Institute of Psychiatry, Psychology and Neuroscience, King's College London, 16 De Crespigny Park, London, SE5 8AB, United Kingdom, 44 7402011448; 2 South London and Maudsley NHS Foundation Trust, London, United Kingdom; 3 NIHR Biomedical Research Centre for Mental Health South London and Maudsley NHS, London, United Kingdom; 4 Department of Psychology, Institute of Psychiatry, Psychology and Neuroscience, King’s College London, London, United Kingdom; 5 School of Life Course and Population Sciences, King’s College London, London, United Kingdom

**Keywords:** atrial fibrillation, mental illness, stroke risk, stroke, clinical decision support systems, CDSS, digital health alerts, decision support, heart, cardiac, arrhythmia, cardiology, interview, qualitative approach, thematic analysis, experience, attitude, opinion, perception, perspective, medical informatics

## Abstract

**Background:**

Electronic clinical decision support systems (eCDSSs) are key to the digital transformation of health care. Despite their growing adoption, little is known about the perspectives of mental health clinicians on the implementation of eCDSS to assist them in managing physical health conditions within mental health care settings.

**Objective:**

This study aimed to explore how clinicians in older adult mental health services manage stroke risk in patients with atrial fibrillation (AF) and comorbid serious mental illness who are admitted to the hospital under their care. It also sought to examine clinicians’ views on the potential role of an eCDSS in enhancing stroke risk assessment and management.

**Methods:**

A cross-sectional mixed methods study was conducted between March and May 2023 in 3 inpatient wards for mental health of older adults at South London and Maudsley NHS (National Health Service) Foundation Trust. Health care professionals, including psychiatrists and pharmacists, participated in a web-based survey and individual semistructured interviews. Ethical approval and informed consent were obtained. A descriptive analysis was conducted on the survey data, while interview data were analyzed thematically using an inductive approach.

**Results:**

In total, 10 clinicians participated in the study. Thematic analysis revealed 2 primary themes. First, clinicians reported significant challenges in clinical practice, including difficulties accessing patient medical histories, limited expertise in managing physical health conditions, fragmented care pathways, and the impact of mental health symptoms such as psychotic beliefs on stroke prevention. Second, clinicians identified strategies to improve practice, such as embedding alerts in electronic records, establishing clear organizational policies, and providing tailored training on AF-related stroke management. Clinicians recognized the potential of an eCDSS to enhance clinical effectiveness, improve the identification of high-risk patients, ensure safer and more consistent care, and save time. However, they expressed concerns about potential risks, including rigidity in decision-making, overreliance on the tool, false positives, reduced critical thinking, annoyance, and increased workload.

**Conclusions:**

This study highlights the challenges and opportunities in managing AF-related stroke risk in mental health settings. While clinicians acknowledged the potential of an eCDSS to improve care quality and efficiency, addressing concerns about its design and implementation is essential. These insights can inform the development of eCDSS tools that effectively balance benefits with user needs, ultimately improving patient outcomes in mental health services.

## Introduction

Electronic clinical decision support systems (eCDSSs) are software-based tools that analyze patient data locked in electronic health records (EHRs) and provide clinicians with relevant clinical support in the form of alerts or reminders [1]. Given the increasing volume of clinical information and the rapid advances in the field of medicine, eCDSSs can be pivotal in providing evidence-based clinical guidelines and tailored clinical support with personalized guidance for diagnostic, therapeutic, and preventive interventions [1].

eCDSSs have gained substantial attention in recent years for their potential to assist health care professionals in selecting appropriate treatment, managing medication (eg, dosing, contraindications, potential interactions, and side effects), calculating risk scores, identifying patients at risk, tracking patient progress over time, and documenting clinical data [2]. This has the potential to reduce medical errors and enhance health outcomes. However, eCDSSs can also have drawbacks. Alert fatigue can result in health care professionals becoming desensitized to notifications and potentially missing important information. This is often the case when the digital tool is overused or poorly designed [2-4]. Clinicians can report feeling overwhelmed with the volume and frequency of alerts, which may, in turn, disrupt workflow, resulting in less face-to-face time with patients [2-4]. Additionally, eCDSSs can lead to incorrect recommendations if the data input are inaccurate or of poor quality [2-4].

Many eCDSSs have been developed to help health care professionals manage physical health conditions, including atrial fibrillation (AF) and associated stroke risk [5]. AF is an arrhythmia characterized by irregular heartbeats. AF disrupts the ability of the heart to pump blood effectively, resulting in a higher risk of blood clotting within the left atrium of the heart and an increased risk of stroke [6]. Based on the National Institute for Health and Care Excellence (NICE) guidelines, patients with AF should undergo a stroke and bleeding risk assessment using the CHA_2_DS_2_-VASc and ORBIT (Outcomes Registry for Better Informed Treatment of Atrial Fibrillation) scales, respectively. NICE recommends oral anticoagulation (OAC) therapy for patients with a CHA_2_DS_2_-VASc score ≥2 and asks clinicians to consider anticoagulation for males with a CHA_2_DS_2_-VASc of 1. When the bleeding risk is low (ORBIT score <3), OAC therapy can be initiated or continued; however, when the risk is moderate or high, careful consideration of the benefits and potential risks associated with the therapy is required [7].

Research assessing the prevalence of AF among people with mental disorders is scarce. A recent nationwide population-based study reported that the risk of AF increased by 2-fold in patients with bipolar disorder or schizophrenia and by 1.5‐ to 1.7-fold in those with depression, insomnia, and anxiety disorders compared to controls [8]. Additionally, people living with a mental illness are at increased risk of cardiovascular disease (including strokes), mainly due to risk factors such as obesity, smoking, diabetes, hypertension, and dyslipidemia [8]. Despite evidence supporting the benefits of OAC therapy, people with AF and comorbid mental health conditions are less likely than the general population to be prescribed OAC therapy [9].

While many studies have evaluated the feasibility, acceptability, and effectiveness of eCDSSs in supporting the management of AF and related stroke risk in general acute hospital settings, these studies were not conducted in mental health care settings [10-19]. Implementing an eCDSS that screens for the stroke risk among patients with AF admitted to a mental health hospital is key to early prevention and quality of life improvement.

This study focuses on older adult mental health services to investigate how AF-related stroke risk is managed in individuals with comorbid severe mental illness, addressing gaps in care to improve outcomes in this high-risk population. Specifically, the study aims to explore (1) clinicians’ experiences in managing AF-related stroke prevention in secondary mental health care services and (2) their perspectives on the potential impact of an eCDSS in enhancing the quality of care in these settings.

## Methods

### Design

This cross-sectional study used a mixed methods research design, incorporating a short web-based survey and individual semistructured interviews.

### Ethical Considerations

Ethical approval was granted by the King’s College London Research Ethics Committee, SLaM Capacity and Capability (Trust R&D Reference: R&D2023/004) and NHS (National Health Service) Health Research Authority (22/HRA/5452). The study was conducted in accordance with the principles of the Declaration of Helsinki (1996) and all applicable regulatory requirements, including but not limited to the UK policy framework for health and social care research, Trust and Research Office policies and procedures, and any subsequent amendments. Information gathered in this study was kept confidential and managed based on the Data Protection Act, NHS Caldicott Guardian, The Research Governance Framework for Health, and Social Care and HRA Approval. Informed consent was obtained from all participants before data collection. Potential participants were provided with an information leaflet outlining the study’s purpose, procedures, and their right to withdraw at any time without consequence. Participants were informed that their participation was voluntary. To ensure privacy and confidentiality, all participant data were anonymized and de-identified. Personal identifiers were removed from the transcripts, and the data were stored securely in compliance with data protection regulations. Any information that could potentially identify participants was excluded from the analysis and reporting. No compensation was provided to participants in this study, as the nature of the study did not involve any direct financial incentives for participation.

### Recruitment

The study was conducted between March and May 2023 in 3 mental health of older adult (MHOA) inpatient wards at South London and NHS Foundation Trust (SLaM). These wards provide specialized care for older adults with a range of mental health conditions, often coexisting with physical health challenges. Purposive sampling was used to identify and recruit participants who were likely to provide clinical care for patients with AF and a comorbid mental health condition.

Senior management on potential wards were first approached by the research team and given brief information regarding the nature of the study and the eCDSS to be implemented. Wards that expressed an interest in the study were provided with further detailed information.

A subgroup of health care professionals, including psychiatrists and pharmacists working on recruited wards, were all invited to complete a short survey and take part in an individual interview. Potential participants varied in terms of professional seniority and clinical experience, with a focus on including diverse perspectives to enrich the findings. Potential participants were given an information leaflet and an opportunity to ask and discuss any further concerns regarding the study. If in agreement to enroll, participants were asked to sign a consent form. The number of participants required for this study was not pre-estimated and was fully dependent on theme saturation in the qualitative part.

### Intervention

The eCDSS consists of a visual prompt integrated into the EHR, which is triggered whenever a patient with documented AF, either chronic or newly diagnosed upon admission, is admitted to the hospital. Using natural language processing, the system identifies references to AF in clinical notes and alerts clinicians to confirm the presence of AF, complete clinical assessments of stroke and bleeding risks using the CHA_2_DS_2_-VASc and ORBIT scales, and record the scores in the EHR. For patients found to have a high risk of stroke, clinicians are prompted to refer them to OAC clinics for specialized care.

### Data Collection

All participants were asked to complete a short web-based survey designed to gather demographic and professional background information, including their age, gender, professional background, and years of clinical experience. The questionnaire was developed to assess clinicians’ awareness and confidence regarding AF-related stroke prevention. It included a series of statements related to their knowledge of AF guidelines, their confidence in assessing stroke and bleeding risks using the CHA_2_DS_2_-VASc and ORBIT scales, and their confidence in managing patients at risk of stroke. Each statement was rated on a Likert scale, ranging from strongly disagree to strongly agree (Multimedia Appendix 1). Example statements included the following: “I am confident in identifying atrial fibrillation patients eligible for oral anticoagulation therapy,” “I am confident in assessing the stroke risk using the CHA_2_DS_2_-VASc tool and the bleeding risk using the ORBIT tool,” and “I am confident in managing atrial fibrillation-related stroke risk in mental healthcare settings.”

The development of the questionnaire involved collaboration with field experts, including psychiatrists, general practitioners (GPs), and health care professionals with expertise in stroke prevention and mental health care. This ensured that the items were relevant to the clinical context and aligned with current guidelines for stroke prevention in patients with AF. The questionnaire was pretested with a small sample of health care professionals to ensure clarity and relevance, and minor revisions were made based on their feedback.

In addition to the survey, an interview schedule was created to explore participants’ experiences with AF-related stroke prevention in secondary mental health care services and the potential impact of an eCDSS on clinician-led care in MHOA wards. The interview topic guide was informed by feedback from field experts, ensuring that the questions were comprehensive and aligned with the research objectives (Multimedia Appendix 2).

Participants were contacted via email, and interviews were scheduled according to their availability. The interviews were conducted via Microsoft Teams, with the same researcher (DF) leading all interviews. Each interview lasted approximately 20 minutes and followed a semistructured format with key prompts to direct the discussion while allowing flexibility for participants to share their insights. All interviews were audio-recorded, transcribed verbatim, and deidentified prior to analysis.

### Data Analysis

Data collected through the questionnaire were analyzed descriptively. Responses to Likert-scale items were summarized to capture the distribution of confidence levels, perceptions of current care quality, and attitudes toward the eCDSS. Demographic and professional background data were also summarized to contextualize participants’ responses.

Thematic analysis was conducted following Braun and Clarke’s framework, incorporating updated guidance from their 2023 work on good practices in thematic analysis [20]. An inductive, data-driven approach was used to allow the themes to emerge directly from the data. The analysis was conducted by 2 members of the research team (DF and HC) and involved several iterative steps. First, both researchers immersed themselves in the data by reading and rereading the transcripts to gain a comprehensive understanding of the content. Descriptive codes were independently generated for each transcript by both researchers, with codes refined and adjusted during subsequent readings. A coding framework was then collaboratively developed based on the descriptive codes, and this framework was iteratively revised to ensure alignment and accommodate different perspectives.

Codes were grouped into broader themes that reflected significant patterns in the data and addressed the research questions. This process involved exploring similarities and differences within and across transcripts and examining patterns based on participant characteristics. Themes were iteratively refined, defined, and labeled to ensure clarity, coherence, and alignment with the data. To enhance the credibility of the findings, the themes were discussed with clinical experts and refined based on their input.

### Reflexivity and Methodological Rigor

The research team actively engaged in reflexivity throughout the study to address potential biases. One of the researchers (DF) brought substantial expertise in applied health informatics and the clinical implications of stroke prevention, which could have shaped their perspective during data collection and analysis. To mitigate this, the researcher frequently reflected on preconceptions and assumptions, documented potential biases, and maintained an ongoing record of decisions made during the research process. Additionally, a second researcher (HC) independently conducted parallel analyses to provide an alternative lens and challenge interpretations.

Transparency and rigor were further enhanced by explicitly acknowledging positionality and engaging clinical experts in the refinement of themes. Methodological rigor was addressed by ensuring confirmability through the maintenance of a clear audit trail of the research process. Dependability was established through the use of a systematic and replicable analytic approach. Credibility was supported by triangulation between researchers and consultation with clinical experts, while transferability was facilitated by providing a detailed description of the study context and participants to enable readers to assess the applicability of the findings to similar settings.

## Results

The sample comprised 10 participants (from a total of 15 invited clinicians), of whom 6 reported their gender as female and 4 as male. Participants’ ages ranged between 25 and 46 years, with a mean age of 32 years. In terms of professional background, a slightly larger number were psychiatrists, which included 3 participants at the consultant level and 3 at a more junior level. The remaining participants (n=4) were pharmacists. The mean years of clinical experience (defined as years a health care professional has spent in clinical practice since professional qualification) was 7.25.

In total, 50% of participants (n=5) considered that AF-related stroke prevention is suboptimal on the wards where they work. Half of participants reported being confident or somewhat confident in managing AF-related stroke prevention in mental health care settings or in making referrals to OAC clinics. Around 60% reported being confident or somewhat confident using the CHA_2_DS_2_-VASc tool to assess the risk of stroke, whereas only 30% reported being confident or somewhat confident using the ORBIT tool to assess the risk of bleeding. Almost all participants strongly agreed that having access to an eCDSS would help them to better assess stroke and bleeding risks in patients with AF.

**Figure 1.**
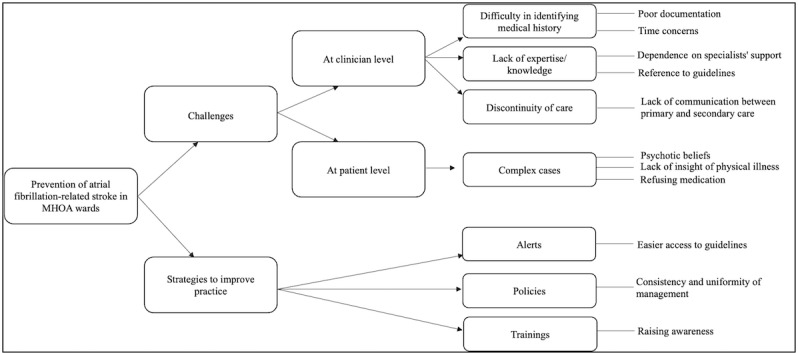
Coding tree of themes pertaining to the prevention of AF-related stroke in mental health of older adult (MHOA) ward.

**Figure 2.**
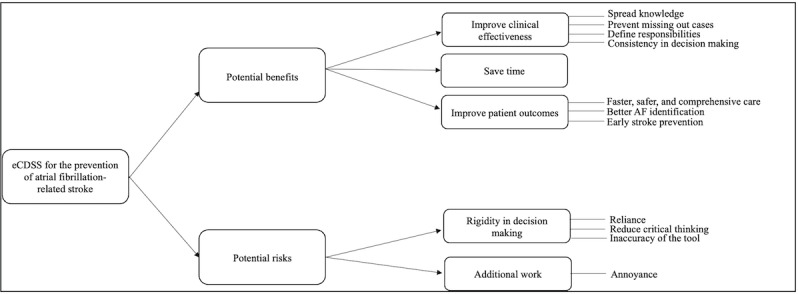
Coding tree of themes pertaining to the potential impact of an electronic clinical decision support system (eCDSS) for the prevention of atrial fibrillation (AF)–related stroke in mental health of older adult wards.

Thematic analysis of the interviews identified 2 overarching themes related to prevention of AF-related stroke: (1) challenges faced on wards and (2) strategies needed to improve practice (Figure 1). As for the potential impact of an eCDSS in improving quality of care, two themes emerged: (1) perceived benefits and (2) perceived risks (Figure 2).

### Prevention of AF-Related Stroke in MHOA Wards

#### Challenges

Participants discussed challenges in the prevention of AF-related stroke in MHOA wards at 2 levels.

At the clinician level, many participants reported that identifying a medical history of AF from the electronic clinical notes is a challenging and time-consuming task. Some of them attributed this to poor documentation of physical health conditions in mental health care settings. Another challenge is clinician lack of knowledge and expertise in the management of physical conditions. To optimize management of physical long-term conditions, most clinicians would seek support from specialists or refer to guidelines such as NICE. Discontinuity of care provision and lack of communication between primary and secondary care were also considered obstacles in the prevention of AF-related stroke in MHOA wards. Participants expressed their concerns about the lack of coordination and follow-up with GPs and its effect on the quality of care (Figure 1 and Table 1).

**Table 1.** Illustrative quotations for the identified subthemes.

Subthemes	Examples
Difficulty in identifying medical history	“sometimes it’s a bit more difficult to get the appropriate history like have they had any previous strokes, do they have any comorbidities, or do they have any other risk factors, family history of stroke.” “The recording of ECG in the department sometimes isn’t great. Uploading ECG onto our clinical document system doesn’t happen a lot of the times, so one of the challenges is to find the ECG initially for people to identify it.” “the doctor usually will read the clinical notes to find out the cardiac history for the patient. This is how we usually identify AF or any stroke history for patient. I guess the challenge is that it’s a bit time wasting because the doctors will have to review previous clinical letters or any discharge summary.”
Lack of expertise or knowledge	“I don’t feel very confident at all, to be honest. I did my foundation years ago and like mental health placement before starting psychiatry training. You know, I used to deal with it a bit on the medical take, but I think if I were to identify a new AF on admission, I’d just discuss it with specialists. But independently, if you were like, sort this out yourself I wouldn’t feel confident.” “Sadly not knowledgeable, I would immediately go and look up the NICE guidance to see the most up to date guidelines because we don’t use it all the time. I probably know when to be worried. I would know where to find the information. But it wouldn’t be all in my mind. But I wouldn’t say I’m knowledgeable at all.” “Well, I’m aware of the CHA2DS2VASc and ORBIT, but not familiar with their use.” “I’d speak to my medical colleagues or end up looking at guidelines and trying to follow because I wouldn’t be kind of regularly checking on what the latest guidance is. So it’d be something I’d have to refresh myself when the situation comes up.”
Discontinuity of care	“I also think the communication between primary and secondary care is probably one of the biggest obstacles.” “The main obstacle I think is the discontinuity of care between secondary care and primary care and also from our side we’re a bit of a like mental health setting so when we’re starting medications that might increase the bleed risk, I think that’s something that we don’t have much of a process for here.”
Complex cases	“Patients refuse to take medications because of their psychotic beliefs or them just having given up, depression, wanting to die, basically, so they don’t have the will to get better.” “A lot of people at this stage of their dementia lack insight to their physical health.” “If we assess the patient to have a high risk of stroke and we want to start on anticoagulants, a lot of our patients actually refuse medication.” “Patients don’t want any of the medication either. They think we’re trying to poison them.” “Patients not taking their medication is quite a common scenario on my ward.”
Alerts	“having PJS alerts is very key, so something that would prompt people and you know provide a very easy pathway for them to follow the guidance rather than kind of spending time to look things up and then not knowing if it’s accurate or if it’s appropriate.” “Reminding people and having these tools easily accessible, you know, so they don’t have to look them up so that they’re incorporated probably in the notes.” “So I think like very concise and clear guidelines. And probably like a hyperlink to where you can do the CHA2DS2VASc and ORBIT scoring and it would then maybe have the kind of action points for the outcome scores. “I think more information. I think what would be good obviously is an explanation on why that’s being recommended, just so that physicians are aware. It’d be great if they could say like well, if the CHA2DS2VASc is greater than this much in these patients, we recommend that, and for example, if it is recommended that they just get monitored annually. Just a statement saying why.” “ So again I I guess the hardest thing with the AF question is that you end up with the CHA2DS2VASc and ORBIT scores or whatever, which is fine. However, you might end up with somebody who you know they score high on CHA2DS2VASc, but then high for ORBIT as well. They obviously have something that flags on, but you don’t really know what that means. There’s still a judgment involved, I guess. So if it was to help with making that part of the Judgment, that I guess would be helpful.” “I think it would be worth including the e-mail address or way to refer to cardiology and everything you need to action that request. What I think would be most useful is kind of like how to do it, what you’re supposed to do with it and how to do what you’re supposed to do with it?” “Well, obviously, where to refer if you need to. Where to refer if you need support or help. And maybe also some contact for local OAC clinics and know where to refer people depending on their GP or their home address or their hospital location. Just so we’re not kind of running around and we just got kind of single referral point of access.”
Policies	“maybe just having like policies on how to manage AF and sort of guidance. I know we have like the physical health guidelines here, but yeah, like a clear pathway would be great.” “I think having some uniformity of how we address things across different wards would be helpful and would provide consistency.” “at trust level, I guess having a policy. Because at SLaM we have a bit of a problem in that we don’t have policies for general health conditions, so I always get calls about where’s SLaM’s policy for that specific physical health conditions.”
Trainings	“At individual level, probably training for our junior doctors and the rest of the team as well. Yeah, training on kind of recognition, and latest guidelines. Also as I mentioned, kind of what to do in a typical situation which you know we might come into contact with our patient cohort.” “I guess it would be just kind of ongoing training to make sure that we are up to date with guideline changes and things.” “I think improving clinicians knowledge of how to manage it. So more awareness of when anticoagulant has to be indicated, how to manage people on anticoagulants. So yeah, just knowledge and like teaching sessions would be great.” “Perhaps during the induction process, this is one of those things that Drs have to be inducted in the expectation that these are the steps that we need to take if somebody does have AF.”
Improve clinical effectiveness	“And then another benefit would be increasing awareness in clinicians about strokes and prevention of strokes especially in elderly patients where these are more common.” “Prompting clinicians and also alerting them can make people feel comfortable, knowing that they’ve got, like, some sort of system in place and like everyone know where the responsibilities lie in terms of managing.” “I mean, I think if it’s consistent, if it’s done for all patients, then we’ll pick up more patients or less will be missed whether or not patient gonna be compliant is the different thing. But at least we’ll pick them up and an attempt to sort of preventing stroke will be made.”
Save time	“The benefits would be to identify, you know information that we want to quickly.” “I guess because it’s hard for clinicians to keep track of the patients or like all the patients all the time, I guess it will help to speed up like to help their job a little bit. When they get notified, they can further look into it rather than missing it out completely.” “the benefits can help you achieve something or kind of assessment risks and benefits and things a bit more quickly.” “Then, certainly it would sort of take into account all the guidelines at the same time and point you in the right direction, which just makes you save time and effort.”
Improve patient care	“Starting prevention and treatment earlier.” “The benefits would be that people are appropriately anticoagulated and we avoid strokes especially that we’ve got lots of people with kind of high physical health care morbidities and vascular risk factors.” “Obviously I think it will reduce the number of patients who might not be getting the appropriate treatment or the appropriate prevention, so that would be the main benefit.” “I guess they can help to prioritise workload for them and it will also highlight physical health problems and I think it will help them to make the decision with a more like a well-rounded approach like considering the physical health factors, not just the mental health.” “So enabling better patient care, faster, maybe more comprehensive, maybe just safer basically if it’s flagging things up.” It “will improve the safety in a tremendous amount to be honest”
Rigidity in decision-making	“I think the main thing is that people can just become kind of blinkered or rigid in their decision-making and kind of forget about the the specific individual factors for that patient that may be quite relevant, but don’t necessarily come up on the on the tool.” “we can start to think that’s the only thing that matters. So like with AF preventing stroke they might just care about the CHA2DS2VASc and ORBIT scores and see what the decision tool makes and they might not be looking at what other things are happening with the patients” “as long as it’s a suggestion and It’s not going to prevent sort of clinical decision-making, it is fine. And I think we need to make sure that yes, it is a prompt and everything but at the end of the day the clinician has to make a decision based on what they believe is appropriate even if it’s not exactly what the tool says. It should be fine as long as we don’t take the thinking out of it and it’s sort of like a tool rather than mandatory in a sense.” “You know automated system can never replace a human you know, because the human person takes into account the individual with their specific circumstances. So most people will probably fit into that system, but there will be others that require more individualized approach.” “there may be mistakes I guess from the electronic system and identifying the wrong thing or misleading us. And I worry that maybe at some point clinicians will just think that if it’s not been highlighted to me electronically, I don’t need to think about it. I think there’s always a danger of that for anything so.” “if the electronic system has any fault to it, then they could potentially lead to a mess.” “Uh harms of this system would be over reliance of electronic systems, we can become a bit over relying I think. A bit of an overreliance sometimes isn’t great.” “It might cause dependency. Clinicians could be just relying on the screening of the electronic system rather than themselves reading into the history.” “I think maybe the disadvantages are that clinicians will be relying on electronic decision support system rather than thinking for themselves or trying to find their information”
Additional work	“There’s lots of things already that we have to do on ePJS and another form is likely, unless it’s really prompting, it’s likely to get forgotten and avoided actively or found to be quite annoying.” “The harm is I don’t know how the tool is. If it’s going to pick up, if it’s going to be accurate and picking up what it picks up, if it’s going to end up more work for the NHS because they’re scrolling through lots of data.” “I guess maybe more paperwork.” “because there’s no more time in the day, you know, like there are sort of limits within which these things are being introduced and It’s like when you’re filling in that new form you are not doing something else and whatever that may be.”

At the patient level, patients with mental illness admitted to MHOA wards are complex, generally having both physical and mental health diagnoses. Illness-related symptoms (eg, delusional beliefs) and active features of illness may result in patient denial of being physically ill, saying that they want to die or refusing medication (Figure 1 and Table 1).

#### Strategies to Improve Practice

To improve AF-related stroke prevention in MHOA wards, most participants suggested sending alerts to clinicians on the patient EHR containing the latest guidelines, including tools for stroke and bleeding risk assessment, guidance on how to interpret the scores, and guidance on how to refer patients at high risk of stroke to OAC clinics. Although most of the information is available online, health care professionals highlighted the importance of making it easily accessible when needed to increase efficiency. They also suggested having policies at the system level for AF-related stroke management to ensure consistency and uniformity in health care provision. At a more individual level, training sessions for health care professionals on the management of AF and how to perform stroke and bleeding risk assessments based on the latest guidelines were thought helpful (Figure 1 and Table 1).

- Subject 9 (psychiatrist): “So I think like very concise and clear guidelines. And probably like a hyperlink to where you can do the CHA_2_DS_2_-VASc and ORBIT scoring and then maybe have the kind of action points for the outcome scores.
- Subject 4 (pharmacist): “maybe just having like policies on how to manage AF and sort of guidance. I know we have like the physical health guidelines here, but yeah, like a clear pathway would be great.”

### eCDSS for the Prevention of AF-Related Stroke

#### Potential Benefits

Most health care professionals reported that an eCDSS for the prevention of AF-related stroke in MHOA wards would improve clinical effectiveness. This could be through spreading knowledge on the management of the condition among clinicians specialized in mental health, defining responsibilities, and ensuring consistency in decision-making. Additionally, participants emphasized the effectiveness of the tool in saving time and speeding up the clinical assessment process. They also reported that an eCDSS would be helpful in improving patient health outcomes as it will ensure faster, safer, and more comprehensive care; improve AF identification in MHOA wards; reduce the chances of getting inappropriate treatments; and ensure early stroke prevention (Figure 2 and Table 1).

- Subject 4 (pharmacist): “Prompting clinicians and also alerting them can make people feel comfortable, knowing that they’ve got, like, some sort of system in place and like everyone know where the responsibilities lie in terms of managing.”
- Subject 3 (psychiatrist): “the benefits can help you achieve something or kind of assessment risks and benefits and things a bit more quickly.”
- Subject 7 (pharmacist): “So enabling better patient care, faster, maybe more comprehensive, maybe just safer basically if it’s flagging things up.”

#### Potential Risks

While an eCDSS can be a very helpful tool for health care professionals, it may have potential risks; one of these is the rigidity in decision-making. Participants reported that they may become overreliant on such digital tools, which may influence their critical thinking skills. They also emphasized that errors in the accuracy of the tool may be misleading and could result in wrong recommendations. Participants were kind of worried about the increased workload caused by the digital tool and reported that annoyance and alert fatigue could be other downsides (Figure 2 and Table 1).

- Subject 3 (psychiatrist): “I think the main thing is that people can just become kind of blinkered or rigid in their decision-making and kind of forget about the specific individual factors for that patient that may be quite relevant, but don’t necessarily come up on the on the tool.”
- Subject 4 (pharmacist): “Uh harms of this system would be over reliance on electronic systems, we can become a bit over relying I think. A bit of an overreliance sometimes isn’t great.”
- Subject 6 (pharmacist): “if the electronic system has any fault to it, then they could potentially lead to a mess.”
- Subject 2 (psychiatrist): “There’s lots of things already that we have to do on ePJS and another form is likely, unless it’s really prompting, it’s likely to get forgotten and avoided actively or found to be quite annoying.”

## Discussion

### Principal Findings

This was an exploratory study that sought to understand mental health care professionals’ experience in the prevention of AF-related stroke and their perspective on the potential impact of an eCDSS in improving that experience. Clinicians reported many challenges related to stroke prevention in MHOA wards, including difficulty identifying patient pertinent medical history, perceived lack of knowledge and expertise in the management of physical conditions, fragmented medical care, and patient psychotic beliefs. To improve clinical practice, they suggested reminding clinicians of the latest guidelines through alerts on patient electronic records, having clear policies at the system level, and providing clinicians with training sessions on AF-related stroke management. Clinicians reported many potential benefits for the eCDSS, including improving clinical effectiveness, better identification of patients at risk, safer and more comprehensive care, consistency in decision-making, and saving time. However, they noted that the digital tool could have potential risks such as rigidity in decision-making, overreliance, reduced critical thinking, false positive recommendations, annoyance, and increased workload.

### Comparison to Prior Work

Physical comorbidities among people with mental illness present complex clinical scenarios that require a specialized and holistic approach to care. Fragmentation between primary and secondary health services could contribute to uncertainty regarding which provider is responsible for the management of physical conditions among people with mental illness [21]. This could result in missed opportunities for the identification of physical conditions, which may be hampered by often poor(er) documentation in mental health services [21]. Additionally, inadequate training and lack of physical care skills may reduce mental health care professionals’ confidence in managing physical conditions [22]. Continuous training, access to resources, and specialist support are all factors that may influence the level of confidence in dealing with acute conditions considered out of their specialty [22]. Another common scenario that prevents or delays the management of physical conditions among people with mental illness is diagnostic overshadowing, which refers to the misattribution of physical symptoms to mental illness [23]. Features of the mental illness itself may also create major challenges, as people experiencing cognitive impairment, hallucinations, or delusions may not recognize or have difficulty communicating symptoms, may resist medication or struggle with medication adherence [23].

The impact of eCDSSs on AF knowledge, OAC prescription, adherence to guidelines, and patient outcomes has been investigated in general health care settings [10-19], with mixed findings on their effectiveness [10-19]. Research aiming to understand clinician perception of how an eCDSS can be supportive and useful is scarce, although this could serve as a basis for creating digital health tools that are impactful and aligned with their needs. In a study conducted in China to evaluate the acceptance of an eCDSS that automatically assesses the risks of stroke and bleeding and suggests treatments accordingly, GPs showed positive attitudes toward the digital tool, reporting that it would be helpful and would strengthen their confidence and capabilities in managing patients with AF [24]. This is consistent with results of our study, where clinicians expressed a lack of confidence in managing stroke risk related to AF and their need to refer to guidelines or to seek advice from specialists even if they already knew about current recommendations. Thus, implementing an eCDSS providing the latest guidelines, tools required to complete clinical assessments for stroke and bleeding risks, and guidance on how to interpret these scores would decrease dependence on specialist inputs and increase clinical efficiency. Our findings are also in line with those of a recent systematic review aiming to identify barriers and facilitators of using CDSSs by primary care professionals [25]. In this review, the reported benefits of the digital tools were improving quality of care, saving time, facilitating decision-making, improving professional self-confidence, and updating knowledge [25]. The main barriers were resistance or reluctance, alert fatigue, information overload, disruption of workflow, negative attitude, lack of motivation to use, lack of computer skills, and validity concerns [25].

### Strengths and Limitations

This study has several strengths. First, it used both quantitative and qualitative data collection and analysis methods, which provided a comprehensive and holistic understanding of the topic of interest. While the quantitative methods offered numerical data, the qualitative approach allowed for a deeper exploration of clinician perceptions and experiences with the digital tool. Second, the study was conducted in 3 wards at South London and Maudsley NHS Foundation Trust (SLaM), which enhances the robustness, applicability, and impact of the research findings in a specific health care context. Third, 2 researchers independently worked on data extraction and analysis, which increased the rigor, transparency, and reliability of the research process. This approach also helped reduce researcher bias and validated the results.

However, the study has several limitations. First, there may have been some reluctance among health care professionals to express their lack of knowledge or confidence in assessing physical health conditions, potentially leading to reporting bias. This was mitigated by explicitly informing clinicians that the data from interviews would be deidentified and that the purpose of the study was to understand their experiences in managing AF-related stroke risk and to inform the implementation of an eCDSS in a helpful way. Second, the study focused only on psychiatrists and pharmacists, as they are typically the professionals involved in clinical assessments related to stroke and bleeding risks. Including other health care professionals with diverse clinical experiences might have enriched the findings and provided a broader perspective. Third, the sample size in this study was relatively small. However, this limitation was addressed by continuing data collection until saturation was reached, ensuring that no new themes emerged from participants’ perspectives. This approach is consistent with findings from a recent systematic review, which suggests that saturation in qualitative research can typically be achieved within 9-17 interviews [26]. Future studies could consider expanding the sample size to confirm the findings and improve the generalizability of the results.

### Conclusions

The study findings indicate that adoption of an eCDSS for stroke risk screening in a psychiatric health service has the potential to be a valuable tool. However, health care organizations and clinicians need to be mindful of the challenges associated with increased workload and the potential overreliance on the system’s recommendations. To maximize the clinical benefits while minimizing the drawbacks, a balanced approach to eCDSS integration is essential. This might involve ongoing training, customization of the system to local practice, and clear guidelines on how to use eCDSS recommendations in conjunction with clinical judgment to provide patient-centered care.

## Supplementary material

10.2196/66428 Multimedia Appendix 1 Clinician survey.

10.2196/66428 Multimedia Appendix 2 Semistructured interview topic guide.

## Abbreviations

AF: atrial fibrillation
eCDSS: electronic clinical decision support systems
EHR: electronic health record
GP: general practitioner
MHOA: mental health of older adult
NHS: National Health Service
NICE: National Institute for Health and Care Excellence
OAC: oral anticoagulation
ORBIT: Outcomes Registry for Better Informed Treatment of Atrial Fibrillation
SLaM: South London and NHS Foundation Trust
