# Supplementary material for: Electronic Clinical Decision Support System for Stroke Risk Screening in Patients With Atrial Fibrillation in Mental Health Care: Mixed Methods Study
Source: JMIR Cardio. 2025 Aug 6;9:e66428. doi: 10.2196/66428 (PMC12327912; doi:10.2196/66428)
Supplement: Multimedia Appendix 1 [file cardio-v9-e66428-s001.docx]

**Appendix 1**


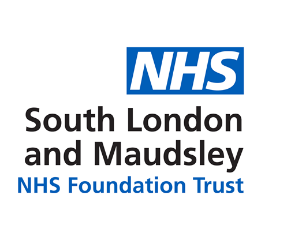

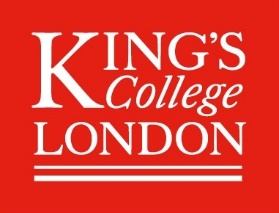


**Implementation of an electronic clinical decision support system (eCDSS) for prevention of atrial fibrillation-related stroke in a mental healthcare setting: a feasibility study**

**Clinician Survey**

**Pre-intervention**

Age:

Gender:

Professional background:

Number of years of clinical experience:

Please rate the degree to which you agree or disagree with each of the following:

|  | **Question** | **Strongly disagree** | **Disagree** | **Somewhat Disagree** | **Not sure** | **Somewhat Agree** | **Agree** | **Strongly Agree** |
| --- | --- | --- | --- | --- | --- | --- | --- | --- |
| 1 | I am aware of guidelines relating to atrial fibrillation-related stroke prevention |  |  |  |  |  |  |  |
| 2 | I am confident in identifying atrial fibrillation patients eligible for oral anticoagulation therapy |  |  |  |  |  |  |  |
| 3 | I am confident in managing atrial fibrillation-related stroke risk in mental healthcare settings |  |  |  |  |  |  |  |
| 4 | I am confident in making referrals to oral anticoagulation clinics |  |  |  |  |  |  |  |
| 5 | I am confident in assessing the stroke risk using the CHA_2_DS_2_VASc tool |  |  |  |  |  |  |  |
| 6 | I am confident in assessing the bleeding risk using the ORBIT tool |  |  |  |  |  |  |  |
| 7 | I am confident in managing bleeding risk factors |  |  |  |  |  |  |  |
| 8 | Having access to an electronic clinical decision support tool (eCDSS)* would help me to better assess stroke and bleeding risks in patients with atrial fibrillation. |  |  |  |  |  |  |  |
| 9 | Atrial fibrillation-related stroke prevention on the ward I work on is currently optimal |  |  |  |  |  |  |  |

**An eCDSS is a health information technology system designed to assist clinicians and other health care professionals in clinical decision-making. In this project, the eCDSS will provide automated CHA_2_DS_2_VASc and ORBIT scores.*
