# Supplementary material for: Electronic Clinical Decision Support System for Stroke Risk Screening in Patients With Atrial Fibrillation in Mental Health Care: Mixed Methods Study
Source: JMIR Cardio. 2025 Aug 6;9:e66428. doi: 10.2196/66428 (PMC12327912; doi:10.2196/66428)
Supplement: Multimedia Appendix 2 [file cardio-v9-e66428-s002.docx]

**Appendix 2**


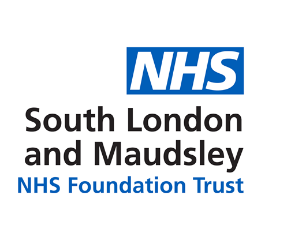

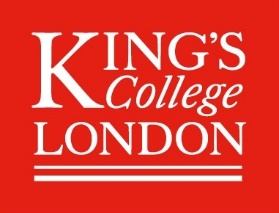


**Implementation of an electronic clinical decision support system (eCDSS) for prevention of atrial fibrillation-related stroke in a mental healthcare setting: a feasibility study**

**Semi structured interview topic guide**

**(Pre- intervention)**

- Exploration of clinician perspectives on atrial fibrillation-related stroke prevention in secondary mental healthcare.

*What challenges do you experience in the prevention of atrial fibrillation-related stroke in this setting? What goes well and what goes less well?*

- Exploration of clinician knowledge of atrial fibrillation-related stroke prevention in secondary mental healthcare.

*How knowledgeable do you consider yourself to be in the prevention of atrial fibrillation-related stroke in this setting?*

*Where do you seek additional guidance when unsure?*

*How knowledgeable do you consider yourself to be when assessing the stroke and bleeding risks in atrial fibrillation patients? Are you aware of any tools for these assessments?*

- Exploration of clinician perspectives of barriers and facilitators to adhering to clinical care guidelines (such as NICE), relating to atrial fibrillation-related stroke prevention.

*Are you aware of any guidelines relating to atrial fibrillation-related stroke prevention? If so, which ones?*

*What do you consider to be the key obstacles to adhering to guidelines for atrial fibrillation-related stroke prevention?*

*What might make it easier for you to adhere to guidelines for atrial fibrillation-related stroke prevention?*

- Exploring clinician perspectives on what might improve atrial fibrillation-related stroke prevention at an individual and wider system level.

*What do you think might help improve atrial fibrillation-related stroke prevention at:*

1. *Individual clinician level*
2. *Wider system level (eg ward or hospital level or Trust level?)*

- Exploring clinician perspectives on use and impact of electronic clinical decision support systems (eCDSSs) in improving clinician led care.

*Do you know what electronic clinical decision support systems are and what they do?*

*Have you ever used an eCDSS? If yes, was it helpful?*

*What potential impact might greater adoption of eCDSSs by clinicians have on patient care?*

*What are the potential benefits and harms of using an eCDSS?*

*What information would be useful to include in an eCDSS for prevention of atrial fibrillation stroke?*
